# Association between the tissue accumulation of advanced glycation end products and exercise capacity in cardiac rehabilitation patients

**DOI:** 10.1186/s12872-020-01484-3

**Published:** 2020-04-23

**Authors:** Mitsuhiro Kunimoto, Kazunori Shimada, Miho Yokoyama, Tomomi Matsubara, Tatsuro Aikawa, Shohei Ouchi, Megumi Shimizu, Kosuke Fukao, Tetsuro Miyazaki, Tomoyasu Kadoguchi, Kei Fujiwara, Abidan Abulimiti, Akio Honzawa, Miki Yamada, Akie Shimada, Taira Yamamoto, Tohru Asai, Atsushi Amano, Andries J. Smit, Hiroyuki Daida

**Affiliations:** 1 Department of Cardiovascular Medicine, Juntendo University Graduate School of Medicine, 2-1-1 Hongo, Bunkyo-ku, Tokyo, 113-8421 Japan; 2 Cardiovascular Rehabilitation and Fitness, Juntendo University Hospital, 2-1-1 Hongo, Bunkyo-ku, Tokyo, 113-8421 Japan; 3 Department of Cardiovascular Surgery, Juntendo University Graduate School of Medicine, 2-1-1 Hongo, Bunkyo-ku, Tokyo, 113-8421 Japan; 4 Division of Vascular Medicine, Department of Internal Medicine, University of Groningen, University Medical Center Groningen, Hanzeplein 1, Groningen, 9713 GZ Netherlands; 5 Faculty of Health Science, Juntendo University, 2-1-1 Hongo, Bunkyo-ku, Tokyo, 113-8421 Japan

**Keywords:** Advanced glycation end products, Exercise tolerance, Cardiac rehabilitation, Skin autofluorescence

## Abstract

**Background:**

Advanced glycation end products (AGEs) are associated with aging, diabetes mellitus (DM), and other chronic diseases. Recently, the accumulation of AGEs can be evaluated by skin autofluorescence (SAF). However, the relationship between SAF levels and exercise capacity in patients with cardiovascular disease (CVD) remains unclear. This study aimed to investigate the association between the tissue accumulation of AGEs and clinical characteristics, including exercise capacity, in patients with CVD.

**Methods:**

We enrolled 319 consecutive CVD patients aged ≥40 years who underwent early phase II cardiac rehabilitation (CR) at our university hospital between November 2015 and September 2017. Patient background, clinical data, and the accumulation of AGEs assessed by SAF were recorded at the beginning of CR. Characteristics were compared between two patient groups divided according to the median SAF level (High SAF and Low SAF).

**Results:**

The High SAF group was significantly older and exhibited a higher prevalence of DM than the Low SAF group. The sex ratio did not differ between the two groups. AGE levels showed significant negative correlations with peak oxygen uptake and ventilator efficiency (both *P* <  0.0001). Exercise capacity was significantly lower in the high SAF group than in the low SAF group, regardless of the presence or absence of DM (*P* <  0.05). A multivariate logistic regression analysis showed that SAF level was an independent factor associated with reduced exercise capacity (odds ratio 2.10; 95% confidence interval 1.13–4.05; *P* = 0.02).

**Conclusion:**

High levels of tissue accumulated AGEs, as assessed by SAF, were significantly and independently associated with reduced exercise capacity. These data suggest that measuring the tissue accumulation of AGEs may be useful in patients who have undergone CR, irrespective of whether they have DM.

## Background

Exercise intolerance is recognized to be an important predictor of adverse outcomes in patients with cardiovascular disease (CVD) [1–3]. Previous studies demonstrated higher mortality rates in patients with heart failure (HF) with reduced exercise capacity (EC), especially in those with peak oxygen uptake (peak VO_2_) ≤14 mL/kg/min [4–7].

Advanced glycation end products (AGEs) are harmful compounds formed when proteins, lipids, and nucleic acids combine with glucose [8]. AGEs accumulate in the body as a result of aging, food intake, and smoking. The reactions that result in AGE accumulation are accelerated under hyperglycemic conditions such as those caused by diabetes mellitus (DM), and in inflammatory conditions, and oxidative stress [9, 10]. AGEs have been shown to directly crosslink proteins, including vascular and muscle collagen, which alters the protein structure and results in dysfunction [8, 11]. Previous studies of the relationship between AGEs and physical function reported that populations with high concentrations of the AGE carboxymethyllysine are more likely to exhibit decreased grip strength and slower walking speed [12, 13].

Skin autofluorescence (SAF) has recently been developed as an accurate and noninvasive method to measure AGE accumulation in the skin. SAF has received attention as its results can provide a useful predictor of all-cause mortality and cardiovascular mortality in patients who are high-risk [14–16].

Evidence that supports that the accumulation of AGEs may be associated with reduced EC exists but whether SAF levels are associated with reduced EC in patients with CVD remains unclear. Thus, the aim of this study was to investigate the association between SAF levels and clinical characteristics in patients with CVD and to evaluate the relationship between SAF levels and EC.

## Methods

### Study population

**Fig. 1:**
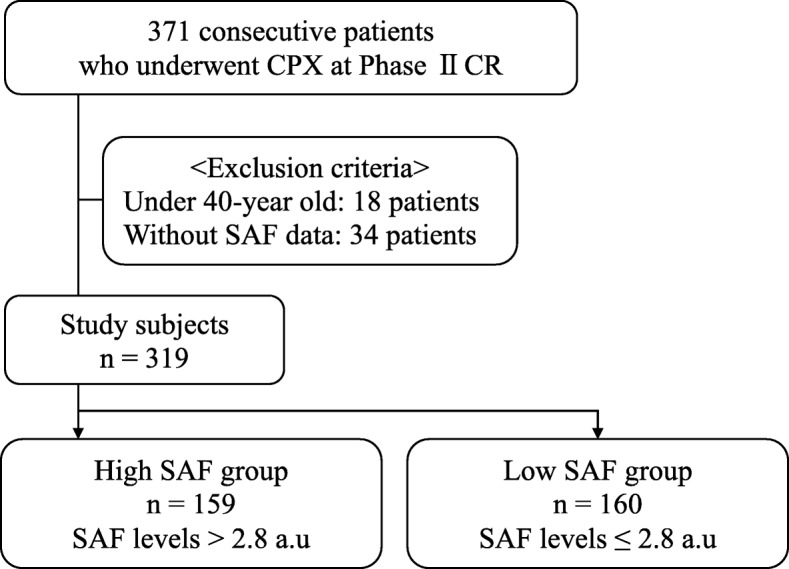
Flowchart of patient enrollment. Consecutive patients who underwent cardiopulmonary exercise testing (CPX) at the beginning of phase II cardiac rehabilitation (CR) were enrolled, totaling 371. The final analysis included 319 patients. CPX, cardiopulmonary exercise test; CR, cardiac rehabilitation; SAF, skin autofluorescence

This retrospective cross-sectional study included 371 consecutive patients who underwent cardiopulmonary exercise testing (CPX) at the beginning of phase II cardiac rehabilitation (CR) at our university hospital between November 2015 and September 2017. Of these, 18 patients were excluded for being aged < 40 years, 34 were excluded because of a lack of SAF data. The final study population consisted of 319 patients (Fig. 1). Written informed consent was provided by all the patients prior to participation. The study protocol was approved by the ethical committee of our institution, and the study was conducted in accordance with the principles of the Helsinki Declaration.

### Skin autofluorescence

SAF levels were measured with an AGE Reader (DiagnOptics Technologies B.V., Groningen, Netherlands) [17]. This noninvasively evaluates the accumulation of AGEs in the skin by measuring the level of fluorescence with light excitation [18]. SAF levels were calculated as the ratio of the average light intensity in the 420–600 nm wavelength range and the average excitation light intensity in the 300–420 nm range. A previous study has shown that AGEs bind and accumulate to collagen and elastin in the epithelium and dermis [19]. A study of healthy and diabetic subjects confirmed that SAF levels assessed by the AGE Reader correlated well with skin biopsy assessments of the accumulation of AGEs such as pentosidine and carboxymethyllysine [20]. Thus, SAF levels provide an indication of the accumulation of AGEs in the epithelium and dermis of the skin. In the present study, SAF was measured from the inside of the forearm while the patient was seated.

### Data collection

Age, sex, smoking history, comorbidities, and medical history were obtained from the patients’ medical records. Blood samples were collected in the early morning after overnight fasting. A diagnosis of DM was defined by hemoglobin A1c ≥ 6.5% or by receiving treatment for DM. Chronic kidney disease (CKD) was defined as an estimated glomerular filtration rate (eGFR) < 60 mL/min/1.73 m2, calculated by the renal disease equation with the Japanese coefficient, using baseline serum creatinine and modification to diet [21].

### Measurements

Body composition, grip strength, SAF level, and EC were assessed at the beginning of CR. Anthropometric parameters, including the percentage of body fat, lean body weight, and muscle mass, were measured by bioelectrical impedance analysis (TANITA, MC-780A, Tokyo, Japan), as described previously [22, 23]. Grip strength was tested in both hands with the patient in a standing position; the higher of the two grip strength values was used in the analysis. EC was assessed by CPX on a cycle ergometer (Strength Ergo 8, Mitsubishi Electric Corp., Tokyo, Japan) with an expiratory gas analysis machine (AE-310S, Minato Medical Science Co., Ltd., Osaka, Japan). A ramp protocol was used with a workload increase of 10 W/min to measure the anaerobic threshold and peak VO_2_. Heart rate was recorded continuously using a standard 12-lead electrocardiogram, and blood pressure was registered every minute during the exercise testing. Peak VO_2_ was defined as the highest VO_2_ value recorded during CPX. The anaerobic threshold point was determined by the V-slope method, as previously described [24]. Patients with a peak VO_2_ ≤ 14 mL/kg/min were categorized as having reduced EC; the other patients were classified as having non-reduced EC.

### Statistical analysis

Continuous variables are presented as mean ± standard deviation. Comparisons between groups were evaluated using Welch’s t test for continuous variables and the chi-squared test for categorical variables. Logistic regression models were used to examine relationships between reduced EC and other factors. We selected covariates with significant differences determined as such by the comparison between the reduced EC and nonreduced EC groups to input into the multivariate analysis. Differences were considered statistically significant at *P* <  0.05. JMP version 12.0 (SAS Institute, Cary, NC, USA) was used to perform the statistical analyses.

## Results

### Baseline characteristics and SAF data

**Fig. 2:**
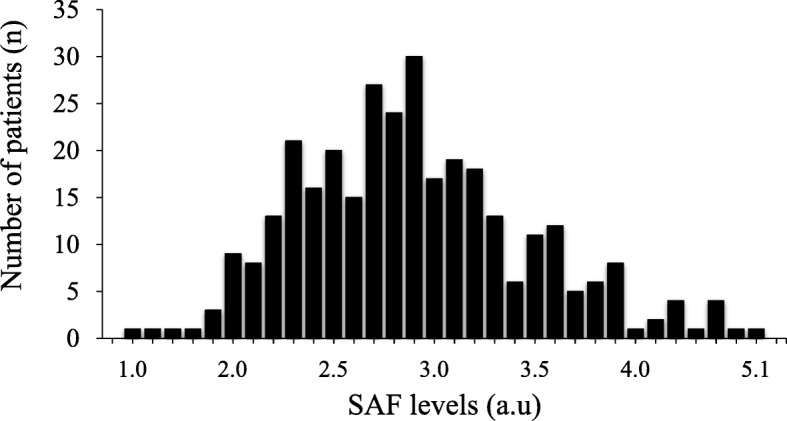
Distribution of SAF levels. The values of mean and median SAF levels were 2.9 ± 0.6 a.u and 2.8 a.u (interquartile range: 2.5, 3.2 a.u), respectively. Shapiro–Wilk test of normality: *P* <  0.05. SAF, skin autofluorescence; DM, diabetes mellitus

In 319 subjects enrolled in the present study, mean age was 66 ± 12 years old, and 256 patients were male (80.3%). Figure 2 shows the distribution of SAF levels. The values of mean and median SAF levels were 2.9 ± 0.6 a.u and 2.8 a.u (interquartile range: 2.5, 3.2 a.u), respectively.

### Comparison between the high SAF and low SAF groups

The patients were divided into two groups based on the median value of SAF. The High and Low SAF groups comprised 159 and 160 participants, respectively. Table 1 compares the clinical characteristics between the two groups. There was no significant difference in sex ratio. Compared to the Low SAF group, the High SAF group exhibited a significantly higher mean age, higher mean body fat percentage, and higher prevalence of DM, CKD, and a history of coronary artery bypass grafting. Cardiac function defined as systolic and diastolic function was similar between the two groups. Hemoglobin and albumin levels were significantly lower in the High SAF group than in the Low SAF group, whereas HbA1c was significantly higher. The anaerobic threshold and peak VO_2_ of the High SAF group were significantly lower than those of the Low SAF group (both *P* <  0.01).

**Table 1:** Patient characteristics

	High SAF(*n* = 159)	Low SAF(*n* = 160)	*P* value
Age	67.9 ± 10.4	60.6 ± 11.8	< 0.01
Male (%)	127 (79.9)	129 (80.6)	0.87
BMI	23.7 ± 3.5	23.3 ± 3.3	0.38
Diabetes mellitus (%)	69 (43.4)	38 (23.8)	< 0.01
Hypertension (%)	107 (67.3)	102 (63.8)	0.51
Dyslipidemia (%)	86 (54.1)	86 (53.8)	0.95
Chronic kidney disease (%)	46 (28.9)	28 (17.6)	0.02
Current smoking (%)	22 (13.8)	22 (13.8)	1
COPD (%)	14 (8.8)	5 (3.1)	0.03
Cancer (%)	0 (0)	3 (1.9)	0.21
History of CVD			
MI (%)	19 (12.0)	16 (10.0)	0.58
PCI (%)	34 (21.4)	22 (13.8)	0.07
CABG (%)	9 (5.7)	6 (3.8)	0.41
Valvular surgery (%)	10 (6.3)	6 (3.8)	0.29
CHF (%)	32 (20.1)	27 (16.9)	0.45
CVD at the beginning of CR			
Acute myocardial infarction (%)	20 (12.6)	19 (11.9)	0.85
Effort angina pectoris (%)	28 (17.6))	20 (12.5)	0.20
PCI (%)	28 (17.6)	25 (15.6)	0.63
CABG (%)	45 (28.3)	24 (15.0)	< 0.01
Valvular disease (%)	58 (36.5)	59 (36.9)	0.94
Valvular surgery (%)	47 (29.8)	50 (31.3)	0.77
Aortic disease (%)	10 (6.3)	13 (8.1)	0.53
Peripheral artery disease (%)	8 (5.0)	3 (1.9)	0.12
Atrial fibrillation (%)	24 (15.1)	26 (16.3)	0.78
Anthropometric data			
Body fat percentage (%)	23.4 ± 7.7	21.4 ± 8.5	0.03
Lean body weight (kg)	48.6 ± 8.4	50.1 ± 8.7	0.13
Trunk muscle mass (kg)	24.8 ± 3.9	25.7 ± 4.2	0.06
Upper limb muscle mass (kg)	4.6 ± 1.0	4.8 ± 1.0	0.15
Lower limb muscle mass (kg)	16.6 ± 4.0	17.1 ± 3.5	0.32
Grip strength (kg)	29.9 ± 8.2	32.3 ± 8.4	0.04
Echocardiography			
LVEF (%)	56 ± 14	57 ± 15	0.74
E/A	1.3 ± 0.9	1.4 ± 0.9	0.42
E/e’	14.1 ± 0.7	13.0 ± 0.7	0.28
Laboratory data			
Hemoglobin (g/dL)	13.3 ± 1.9	13.9 ± 1.7	< 0.01
Albumin (g/dL)	3.9 ± 0.4	4.0 ± 0.5	0.03
Creatinine (mg/dL)	1.11 ± 1.4	0.8 ± 0.3	< 0.01
eGFR (mL/min/1.73 m^2^)	70.1 ± 25.7	77.2 ± 19.4	< 0.01
TG (mg/dL)	114 ± 63	130 ± 87	0.07
HDL cholesterol (mg/dL)	49 ± 15	50 ± 16	0.68
LDL cholesterol (mg/dL)	100 ± 28	100 ± 30	0.88
HbA1c (%)	6.1 ± 0.8	5.8 ± 0.6	< 0.01
BNP (pg/nL)	200.6 ± 516.0	160 ± 287	0.40
Skin autofluorescence (a.u)	3.3 ± 0.4	2.4 ± 0.3	< 0.01
Medication			
Aspirin (%)	130 (82.3)	12 (78.1)	0.35
ACE-I/ARB (%)	66 (41.8)	62 (38.8)	0.58
Statin (%)	106 (67.1)	87 (54.4)	0.02
β blocker (%)	116 (73.4)	116 (72.5)	0.85
Ca antagonist (%)	29 (18.4)	21 (13.1)	0.20
Loop diuretics (%)	110 (69.6)	108 (67.5)	0.68
Oral hypoglycemic agent (%)	35 (22.2)	13 (8.1)	< 0.01
Insulin (%)	14 (8.9)	0 (0)	< 0.01
Anaerobic threshold (AT)			
Workload (W)	43 ± 14	49 ± 15	< 0.01
AT (mL/kg/min)	10.7 ± 2.2	11.8 ± 2.5	< 0.01
Peak exercise			
HR (/min)	111 ± 19	116 ± 20	0.03
SBP (mmHg)	178 ± 30	173 ± 31	0.16
DBP (mmHg)	86 ± 17	87 ± 17	0.41
RER	1.12 ± 0.11	1.11 ± 0.10	0.18
Workload (W)	77 ± 20	86 ± 21	< 0.01
Peak VO_2_ (mL/kg/min)	15.6 ± 3.5	17.2 ± 3.8	< 0.01
VE/VCO_2_	32.4 ± 7.5	29.5 ± 6.5	< 0.01

High SAF; defined as SAF levels > 2.8

Data are presented as the mean value ± SD. *BMI* body mass index, *COPD* chronic obstructive pulmonary disease, *CVD* cardiovascular disease, *MI* myocardial infarction, *PCI* percutaneous coronary intervention, *CABG* coronary artery bypass graft, *CHF* congestive heart failure, *CR* cardiac rehabilitation, *LV* left ventricular, *EF* ejection fraction, *E* early diastolic filling velocity, *A* late diastolic filling velocity, *e’* early diastolic tissue velocity, *eGFR* estimate glomerular filtration rate, *TG* triglyceride, *HDL* high-density lipoprotein cholesterol, *LDL* low-density lipoprotein cholesterol, *HbA1c* hemoglobin A1c, *BNP* B-type natriuretic peptide, *HR* heart rate, *SBP* systolic blood pressure, *DBP* diastolic blood pressure, *RER* respiratory exchange ratio, *peak VO*_*2*_ peak oxygen uptake

### Comparison between the diabetes and non-diabetes groups

**Fig. 3:**
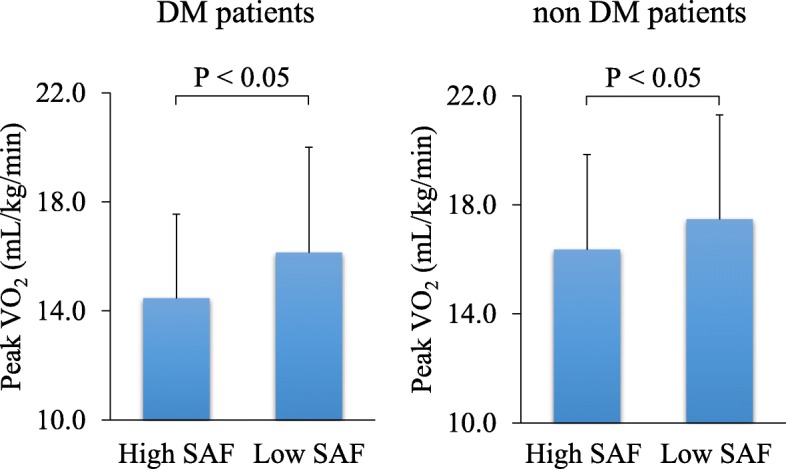
Comparison of peak oxygen uptake (VO_2_) levels between the High (> 2.8 a.u.) and low (≤ 2.8 a.u.) SAF groups. Regardless of the presence or absence of DM, peak VO_2_ levels were significantly lower in the high SAF group than in the low SAF group. SAF, skin autofluorescence; DM, diabetes mellitus

The patients were divided into two groups by DM status and then classified as high or low SAF according to the median SAF level for the group (3.0 a.u. for the DM group and 2.7 a.u. for the non-DM group). Comparisons of the clinical characteristics between the high and low SAF subgroups tended to show the same trends in the DM and non-DM groups (Supplemental Tables 1 and 2). In the DM group, peak VO_2_ was significantly lower in the high SAF subgroup than in the low SAF subgroup (14.5 ± 3.1 vs. 16.1 ± 3.9 mL/kg/min, respectively; *P* <  0.05). Similarly, in the non-DM group, peakVO_2_ was significantly lower in the high SAF subgroup compared to the low SAF subgroup (16.4 ± 3.5 vs. 17.5 ± 3.8 mL/kg/min; P <  0.05) (Fig. 3).

### Association between SAF levels and reduced EC

Reduced EC is considered to be clinically important [4–7]. Therefore, we performed a logistic regression analysis to investigate the factors that were independently associated with reduced EC, defined as peak VO_2_ ≤ 14 mL/kg/min (Supplemental Table 3 shows the comparisons of the clinical characteristics between the reduced and non-reduced EC groups). After adjusting for age, sex, BMI, DM, Atrial fibrillation, body fat percentage, E/e’, albumin, eGFR, HDL-cholesterol, BNP, aspirin and SAF, SAF level was found to be a significant independent factor associated with reduced EC (odds ratio 2.10, 95% confidence interval 1.13–4.05; *P* = 0.02) (Table 2).

**Table 2:** Logistic regression analysis of reduced EC

Variables	Univariate			Multivariate		
	Odds ratio	95% CI	P value	Odds ratio	95% CI	P value
Age	1.05	1.03–1.08	< 0.01	1.04	1.00–1.09	0.04
Female	1.82	1.01–3.22	< 0.05	0.97	0.31–2.96	0.96
BMI	1.08	1.01–1.17	< 0.05	0.98	0.83–1.15	0.76
Diabetes mellitus	2.64	1.61–4.38	< 0.01	1.56	0.74–3.26	0.24
Atrial fibrillation	3.99	2.15–7.52	< 0.01	1.48	0.55–3.90	0.43
Albumin	0.45	0.26–0.77	< 0.01	1.22	0.56–2.72	0.62
eGFR	0.97	0.96–0.99	< 0.01	0.99	0.97–1.01	0.18
BNP	1.00	1.00–1.002	< 0.01	1.00	0.99–1.00	0.73
E/e’	1.06	1.02–1.10	< 0.01	1.05	1.00–1.10	0.04
Body fat percentage	1.09	1.05–1.13	< 0.01	1.07	0.99–1.16	0.054
HDL cholesterol	0.98	0.96–0.99	< 0.05	0.98	0.96–1.01	0.14
Aspirin	0.46	0.26–0.83	< 0.01	0.57	0.20–1.63	0.29
SAF	2.63	1.72–4.13	< 0.01	2.10	1.13–4.05	0.02

*EC* exercise capacity, *CI* confidence interval, *BMI* body mass index, *eGFR* estimate glomerular filtration rate, *BNP* B-type natriuretic peptide, *E* early diastolic filling velocity, *e’* early diastolic tissue velocity, *HDL* high density lipoproteins, SAF skin autofluorescence

## Discussion

The results of this study showed that EC was significantly lower in patients with higher SAF levels regardless of their DM status and that SAF levels were independently associated with reduced EC, even while cardiac systolic and diastolic function were similar between both groups. To the best of our knowledge, this is the first study to demonstrate an association between SAF levels and reduced EC among patients with CVD who underwent CR.

Several studies reported an association between plasma AGEs and lower physical function in community-dwelling elderly people [12, 13]. In addition, a study of a Japanese population reported that SAF levels were significantly higher in the study group with lower muscle mass (defined by the Asian Working Group for Sarcopenia’s skeletal muscle mass index criteria) and were a significant independent factor associated with low skeletal muscle index values [17]. The results of the present study further confirm the relationship between SAF levels and reduced EC. This is an important finding because impaired EC is known to be a powerful predictor of poor prognosis [1–3]. A previous study of patients with HF with systolic dysfunction reported that SAF level were significantly higher and EC was significantly lower in patients with DM than in those without DM [25]. In addition, the patients with SAF levels above the mean value demonstrated lower EC [25]. Our findings are consistent with those of the previous report, and we additionally found that patients with higher SAF levels demonstrated significantly lower EC, even in the patients without DM.

It has been reported that patients with DM showed reduced muscle function and EC [26–28]. Although the determinants of impaired physical function in patients with DM are poorly understood, several mechanisms have been proposed [29]. Previous studies demonstrated an inverse correlation between EC measured by peak VO_2_ and insulin resistance, and that increased SAF levels were positively associated with insulin resistance in patients with DM [30–32]. It has also been reported that serum AGE levels were positively correlated with insulin resistance even in non-DM patients [33]. Furthermore, previous studies reported that diabetes and hyperglycemia are associated with mitochondrial dysfunction and increased levels of mitochondrial reactive oxygen species in the vasculature, resulting in endothelial nitric oxide synthase inhibition [34, 35]. In addition to these direct effects, AGEs can bind with AGE receptors, which can result in endothelial dysfunction and the enhanced production of reactive oxygen species [36]. Animal experiments suggested that endothelial nitric oxide could be a factor in EC regulation [34–37]. Crosslinking of myocardial collagen with AGEs may contribute to increased myocardial stiffness and diastolic dysfunction [11, 36]. In addition, left ventricular diastolic dysfunction due to DM is associated with decreased left ventricular compliance, resulting in a restricted ability to increase cardiac output during exercise, thereby limiting EC [38, 39]. Previous studies have reported that diastolic dysfunction assessed by E/e’ is a strong predictor of exercise intolerance, and this association was independent of DM [40, 41]. Consistent with these studies, our investigation demonstrated that E/e’ was one of the significant factors influencing reduced EC. In the present study, age was also associated with reduced EC. It has been suggested that the effects of aging on exercise intolerance are due in part to decreased activity and changes in body composition [42]. Moreover, the accumulation of AGEs has also been observed to be associated with aging, lifestyle habits such as specific food intake, and smoking, in addition to the presence of chronic inflammatory conditions such as metabolic syndrome, arteriosclerosis, and renal disease [9, 10, 43]. The deterioration of EC with AGE accumulation may, therefore, be the result of AGEs causing the functional decline of several organ systems that regulate EC, regardless of DM status. A recent meta-analysis demonstrated that SAF levels were a predictor of mortality in high-risk patients [15]. This could potentially be explained by the association between high SAF levels and reduced EC observed in the present study. As for interventions, a recent study reported that alagebrium, proposed as an AGE-breaker, did not ameliorate EC and cardiac function, but also failed to lower SAF levels in patients with HF [44]. Thus, further studies are needed to elucidate the mechanisms by which AGEs affect EC.

Body fat percentage also tended to have an effect on reduced EC in the present study. Although interactions between AGEs and adipocytes have not been fully clarified [45], the accumulation of body fat may relate to deterioration in insulin sensitivity, increased intracellular lipids in skeletal muscle, and the decreased metabolic ability of mitochondria, ultimately resulting in decreased oxygen intake during exercise [46–49].

This study exhibits several limitations. First, this was a single-center and retrospective cross-sectional study with a small sample size, so we could not establish a causal relationship between SAF level and reduced EC. Second, we enrolled patients with CVD who underwent CR. Third, the method of SAF assessment did not measure the total accumulation of all AGEs in the body. Fourth, SAF represents not only the fluorescence values resulting from skin AGEs, but also from other fluorophores such as keratin, therefore assessment of SAF may not be an accurate measurement of the skin’s AGE content [50]. Fifth, previous studies suggest that the reliability of AGE analysis in skin may depend on skin color, as this affects the skin’s tendency to absorb excitation light [51]. Sixth, SAF is strongly influenced by the use of skin creams, which leads to elevated SAF values and decreased skin reflectance [52]. Seventh, SAF is a surrogate marker of tissue accumulation of AGEs. Whether skin AGEs reflect the accumulation of AGEs in cardiac tissue is an important question, and further investigations are needed to answer it. Finally, the diagnosis of DM may have been underestimated because some patients did not undergo an oral glucose tolerance test.

## Conclusion

In conclusion, this study demonstrated that high levels of tissue accumulated AGEs, as assessed by SAF, were significantly and independently associated with reduced EC. These data suggest that the measurement of the tissue accumulation of AGEs may be useful for patients undergoing CR, including those without DM. Further studies should be carried out to determine whether elevated SAF levels are a specific predicter of decline in EC in patients undergoing CR and to corroborate these findings in other patients with CVD.

## Supplementary information

**Additional file 1: Table S1**. Comparison of clinical characteristics between High SAF (> 3.0 a. u.) and Low SAF (≤ 3.0 a.u.) groups in DM (diabetes mellitus) patients.

**Additional file 2: Table S**2. Comparison of clinical characteristics between (> 2.7 a.u.) and Low SAF (≤ 2.7 a.u.) groups in non DM (diabetes mellitus) patients.

**Additional file 3: Table S**3. Comparison of clinical characteristics between reduced EC and non-reduced EC groups.

## Data Availability

The datasets used and/or analysed during the current study are available from the corresponding author on reasonable request.

## Abbreviations

CVD: Cardiovascular disease
HF: Heart failure;
EC: Exercise capacity
AGEs: Advanced glycation end products
DM: Diabetes mellitus
SAF: Skin autofluorescence
CPX: Cardiopulmonary exercise testing
CR: Cardiac rehabilitation
CKD: Chronic kidney disease.
