# Supplementary material for: Association between the tissue accumulation of advanced glycation end products and exercise capacity in cardiac rehabilitation patients
Source: BMC Cardiovasc Disord. 2020 Apr 23;20:195. doi: 10.1186/s12872-020-01484-3 (PMC7178950; doi:10.1186/s12872-020-01484-3)
Supplement: Supplementary file 1 — Additional file 1: Table S1. Comparison of clinical characteristics between High SAF (> 3.0 a. u.) and Low SAF (≤ 3.0 a.u.) groups in DM (diabetes mellitus) patients. [file 12872_2020_1484_MOESM1_ESM.docx]

Supplemental Table 1. Comparison of clinical characteristics between High SAF (> 3.0 a. u.) and Low SAF (≤ 3.0 a.u.) groups in DM (diabetes mellitus) patients

|  | High SAF  (n = 49) | Low SAF (n = 58) | P value |
| --- | --- | --- | --- |
| Age | 70.1 ± 9.1 | 64.5 ± 12.7 | 0.01 |
| Male (%) | 43 (87.8) | 46 (79.3) | 0.24 |
| BMI | 24.7 (3.4) | 24.7 (3.4) | 0.95 |
| Diabetes (%) | 49 (100) | 58 (100) | n.s |
| Hypertension (%) | 34 (69.4) | 45 (77.6) | 0.34 |
| Dyslipidemia (%) | 31 (63.3) | 43 (74.1) | 0.23 |
| Chronic kidney disease (%) | 15 (30.6) | 16 (27.6) | 0.73 |
| Current smoking (%) | 6 (12.2) | 10 (17.2) | 0.47 |
| COPD (%) | 8 (16.3) | 1 (1.7) | < 0.01 |
| Cancer (%) | 0 (0) | 0 (0) | n.s |
| History of CVD | | | |
| MI　(%) | 9 (18.4) | 8 (13.8) | 0.53 |
| PCI　(%) | 18 (36.7) | 15 (25.9) | 0.23 |
| CABG (%) | 3 (6.1) | 4 (6.9) | 0.87 |
| Valvular surgery (%) | 3 (6.1) | 4 (6.9) | 0.87 |
| CHF (%) | 9 (18.4) | 19 (32.8) | 0.09 |
| CVD at the beginning of CR | | | |
| Acute myocardial infarction (%) | 5 (10.2) | 7 (12.1) | 0.76 |
| Effort angina pectoris (%) | 12 (24.5) | 8 (13.8) | 0.16 |
| PCI (%) | 8 (16.3) | 11 (19.0) | 0.72 |
| CABG (%) | 25 (51.0) | 11 (19.0) | < 0.01 |
| Valvular disease (%) | 11 (22.5) | 12 (20.7) | 0.82 |
| Valvular surgery (%) | 8 (16.3) | 8 (13.8) | 0.71 |
| Aortic disease (%) | 1 (2.0) | 0 (0) | 0.27 |
| Peripheral artery disease (%) | 4 (8.2) | 3 (5.2) | 0.53 |
| Atrial fibrillation (%) | 9 (18.4) | 17 (29.3) | 0.19 |
| Anthropometric data | | | |
| Body fat percentage (%) | 25.1 ± 8.0 | 24.9 ± 8.0 | 0.90 |
| Lean body weight (kg) | 50.7 ± 8.0 | 49.7 ± 8.5 | 0.53 |
| Trunk muscle mass (kg) | 25.8 ± 3.7 | 24.9 ± 4.3 | 0.25 |
| Upper limb muscle mass (kg) | 5.0 ± 0.9 | 4.8 ± 1.0 | 0.52 |
| Lower limb muscle mass (kg) | 17.4 ± 3.7 | 17.4 ± 3.7 | 0.95 |
| Grip strength | 30.8 ± 6.7 | 29.2 ± 7.1 | 0.32 |
| Echocardiography | | | |
| EF | 57 ± 12 | 54 ± 17 | 0.25 |
| E/A | 1.3 ± 0.7 | 1.5 ± 1.1 | 0.25 |
| E/e' | 14.2 ± 6.7 | 15.4 ± 11.3 | 0.55 |
| Laboratory data | | | |
| Hemoglobin (g/dL) | 13.3 ± 1.5 | 14.1 ± 2.2 | 0.04 |
| Albumin (g/dL) | 3.8 ± 0.5 | 4.0 ± 0.5 | 0.18 |
| Creatinine (mg/dL) | 1.29 ± 1.80 | 0.87 ± 0.37 | 0.09 |
| eGFR (mL/min/1.73 m^2^) | 65.6 ± 25.2 | 73.4 ± 22.0 | 0.09 |
| TG (mg/dL) | 127 ± 60 | 138 ± 103 | 0.50 |
| HDL cholesterol (mg/dL) | 44 ± 11 | 49 ± 17 | 0.09 |
| LDL cholesterol (mg/dL) | 94 ± 24 | 93 ± 28 | 0.77 |
| HbA1c (%) | 6.9 ± 0.8 | 6.6 ± 0.6 | 0.03 |
| BNP (pg/nL) | 170.3 ± 203.3 | 200.5 ± 409.2 | 0.64 |
| Skin autofluorescence (a.u) | 3.6 ± 0.4 | 2.6 ± 0.3 | < 0.01 |
| Medication | | | |
| Aspirin (%) | 41 (83.7) | 43 (74.1) | 0.23 |
| ACE-I/ARB (%) | 21 (42.9) | 37 (63.8) | 0.03 |
| Statin (%) | 39 (79.6) | 41 (70.7) | 0.29 |
| β blocker (%) | 36 (73.5) | 45 (77.6) | 0.62 |
| Ca antagonist (%) | 11 (22.5) | 11 (19.0) | 0.66 |
| Loop diuretics (%) | 32 (65.3) | 37 (63.8) | 0.87 |
| Oral hypoglycemic agent (%) | 26 (53.1) | 22 (37.9) | 0.12 |
| Insulin (%) | 8 (16.3) | 6 (10.3) | 0.36 |
| Anaerobic threshold (AT) | | | |
| Workload (W) | 43 ± 14 | 49 ± 19 | 0.08 |
| AT (mL/kg/min) | 10.0 ± 1.9 | 11.2 ± 2.6 | < 0.01 |
| Peak exercise | | | |
| HR (/min) | 106 ± 20 | 114 ± 21 | 0.07 |
| SBP (mmHg) | 178 ± 30 | 172 ± 35 | 0.35 |
| DBP (mmHg) | 85 ± 17 | 86 ± 18 | 0.86 |
| RER | 1.12 ± 0.11 | 1.11 ± 0.10 | 0.51 |
| Workload (W) | 76 ± 21 | 83 ± 23 | 0.08 |
| Peak VO_2_ (mL/kg/min) | 14.5 ± 3.1 | 16.1 ± 3.9 | 0.02 |
| VE/VCO_2_ | 33.9 ± 7.1 | 29.7 ± 5.7 | < 0.01 |

High SAF; defined as SAF levels >3.0.

Data are presented as the mean value ± SD. BMI, body mass index; COPD, chronic obstructive pulmonary disease; CVD, cardiovascular disease; MI, myocardial infarction; PCI, percutaneous coronary intervention; CABG, coronary artery bypass graft; CHF, congestive heart failure; CR, cardiac rehabilitation; LV, left ventricular; EF, ejection fraction; E, early diastolic filling velocity; A, late diastolic filling velocity; e’, early diastolic tissue velocity; eGFR, estimate glomerular filtration rate; TG, triglyceride; HDL, high-density lipoprotein cholesterol; LDL, low-density lipoprotein cholesterol; HbA1c, hemoglobin A1c; BNP, B-type natriuretic peptide; HR, heart rate; SBP, systolic blood pressure; DBP, diastolic blood pressure; RER, respiratory exchange ratio; peak VO_2_, peak oxygen uptake.
