# Supplementary material for: Association between the tissue accumulation of advanced glycation end products and exercise capacity in cardiac rehabilitation patients
Source: BMC Cardiovasc Disord. 2020 Apr 23;20:195. doi: 10.1186/s12872-020-01484-3 (PMC7178950; doi:10.1186/s12872-020-01484-3)
Supplement: Supplementary file 2 — Additional file 2: Table S2. Comparison of clinical characteristics between (> 2.7 a.u.) and Low SAF (≤ 2.7 a.u.) groups in non DM (diabetes mellitus) patients. [file 12872_2020_1484_MOESM2_ESM.docx]

Supplemental Table 2. Comparison of clinical characteristics between (> 2.7 a.u.) and Low SAF (≤ 2.7 a.u.) groups in non DM (diabetes mellitus) patients

|  | High SAF  (n = 106) | Low SAF (n = 106) | P value |
| --- | --- | --- | --- |
| Age | 66.3 ± 10.8 | 59.3 ± 11.3 | < 0.01 |
| Male (%) | 81 (76.4) | 86　(81.1) | 0.40 |
| BMI | 23.0 (3.4) | 22.8 (3.0) | 0.74 |
| Diabetes (%) | - | - | - |
| Hypertension (%) | 68 (64.2) | 62 (58.5) | 0.40 |
| Dyslipidemia (%) | 50 (47.2) | 48 (45.3) | 0.78 |
| Chronic kidney disease (%) | 26 (24.5) | 17 (16.2) | 0.13 |
| Current smoking (%) | 16 (15.1) | 12 (11.4) | 0.43 |
| COPD (%) | 6 (5.7) | 4 (3.8) | 0.52 |
| Cancer (%) | 0 (0) | 3 (2.8) | 0.21 |
| History of CVD | | | |
| MI (%) | 7 (6.6) | 11 (10.4) | 0.32 |
| PCI (%) | 13 (12.3) | 10 (9.4) | 0.51 |
| CABG (%) | 5 (4.8) | 3 (2.8) | 0.46 |
| Valvular surgery (%) | 5 (4.8) | 4 (3.8) | 0.72 |
| CHF (%) | 15 (14.2) | 16 (15.1) | 0.85 |
| CVD at the beginning of CR | | | |
| Acute myocardial infarction (%) | 16 (15.1) | 11 (10.4) | 0.30 |
| Effort angina pectoris (%) | 17 (16.0) | 11 (10.4) | 0.22 |
| PCI (%) | 20 (18.9) | 14 (13.2) | 0.26 |
| CABG (%) | 20 (18.9) | 13 (12.3) | 0.18 |
| Valvular disease (%) | 44 (41.5) | 50 (47.2) | 0.41 |
| Valvular surgery (%) | 39 (37.1) | 42 (39.6) | 0.71 |
| Aortic disease (%) | 12 (11.3) | 10 (9.4) | 0.65 |
| Peripheral artery disease (%) | 4 (3.8) | 0 (0) | 0.04 |
| Atrial fibrillation (%) | 9 (8.5) | 15 (14.2) | 0.19 |
| Anthropometric data | | | |
| Body fat percentage (%) | 22.0 ± 7.3 | 20.0 ± 8.5 | 0.07 |
| Lean body weight (kg) | 47.9 ± 8.5 | 50.1 ± 8.8 | 0.07 |
| Trunk muscle mass (kg) | 24.6 ± 4.0 | 25.8 ± 4.3 | 0.03 |
| Upper limb muscle mass (kg) | 4.5 ± 1.1 | 4.8 ± 1.0 | 0.09 |
| Lower limb muscle mass (kg) | 16.2 ± 4.0 | 16.9 ± 3.5 | 0.19 |
| Grip strength | 30.8 ± 9.0 | 32.8 ± 8.9 | 0.19 |
| Echocardiography | | | |
| EF | 57 ± 14 | 57 ± 14 | 0.90 |
| E/A | 1.2 ± 0.8 | 1.6 ± 1.0 | 0.01 |
| E/e' | 12.7 ± 6.5 | 13.3 ± 7.6 | 0.57 |
| Laboratory data | | | |
| Hemoglobin (g/dL) | 13.2 ± 1.9 | 13.8 ± 1.5 | 0.03 |
| Albumin (g/dL) | 3.9 ± 0.5 | 4.0 ± 0.4 | 0.18 |
| Creatinine (mg/dL) | 1.03 ± 1.15 | 0.80 ± 0.23 | 0.04 |
| eGFR (mL/min/1.73 m^2^) | 71.8 ± 24.1 | 79.3 ± 20.0 | 0.01 |
| TG (mg/dL) | 107 ± 63 | 127 ± 75 | 0.04 |
| HDL cholesterol (mg/dL) | 51 ± 16 | 50 ± 15 | 0.42 |
| LDL cholesterol (mg/dL) | 104 ± 31 | 103 ± 28 | 0.72 |
| HbA1c (%) | 5.7 ± 0.4 | 5.6 ± 0.4 | 0.14 |
| BNP (pg/nL) | 211.0 ± 619.2 | 144.2 ± 189.1 | 0.30 |
| Skin autofluorescence (a.u) | 3.2 ± 0.4 | 2.4 ± 0.3 | < 0.01 |
| Medication | | | |
| Aspirin (%) | 87 (82.9) | 84 (79.3) | 0.50 |
| ACE-I/ARB (%) | 35 (33.3) | 35 (33.0) | 0.96 |
| Statin (%) | 65 (61.9) | 48 (45.3) | 0.02 |
| β blocker (%) | 73 (69.5) | 78 (73.6) | 0.51 |
| Ca antagonist (%) | 17 (16.2) | 11 (10.4) | 0.21 |
| Loop diuretics (%) | 71 (67.6) | 78 (73.6) | 0.34 |
| Oral hypoglycemic agent (%) | - | - | - |
| Insulin (%) | - | - | - |
| Anaerobic threshold (AT) | | | |
| Workload (W) | 44 ± 13 | 49 ± 15 | < 0.01 |
| AT (mL/kg/min) | 11.2 ± 2.2 | 12.0 ± 2.4 | 0.02 |
| Peak exercise | | | |
| HR (/min) | 114 ± 18 | 115 ± 20 | 0.56 |
| SBP (mmHg) | 180 ± 29 | 172 ± 29 | 0.03 |
| DBP (mmHg) | 86 ± 17 | 87 ± 16 | 0.61 |
| RER | 1.13 ± 0.12 | 1.10 ± 0.10 | 0.11 |
| Workload (W) | 79 ± 20 | 85 ± 21 | 0.03 |
| Peak VO_2_ (mL/kg/min) | 16.4 ± 3.5 | 17.5 ± 3.8 | 0.03 |
| VE/VCO_2_ | 31.2 ± 7.9 | 30.0 ± 6.7 | 0.23 |

High SAF; defined as SAF levels >2.7.

Data are presented as the mean value ±SD. BMI, body mass index; COPD, chronic obstructive pulmonary disease; CVD, cardiovascular disease; MI, myocardial infarction; PCI, percutaneous coronary intervention; CABG, coronary artery bypass graft; CHF, congestive heart failure; CR, cardiac rehabilitation; LV, left ventricular; EF, ejection fraction; E, early diastolic filling velocity; A, late diastolic filling velocity; e’, early diastolic tissue velocity; eGFR, estimate glomerular filtration rate; TG, triglyceride; HDL, high-density lipoprotein cholesterol; LDL, low-density lipoprotein cholesterol; HbA1c, hemoglobin A1c; BNP, B-type natriuretic peptide; HR, heart rate; SBP, systolic blood pressure; DBP, diastolic blood pressure; RER, respiratory exchange ratio; peak VO_2_, peak oxygen uptake.
