# Supplementary material for: Association between the tissue accumulation of advanced glycation end products and exercise capacity in cardiac rehabilitation patients
Source: BMC Cardiovasc Disord. 2020 Apr 23;20:195. doi: 10.1186/s12872-020-01484-3 (PMC7178950; doi:10.1186/s12872-020-01484-3)
Supplement: Supplementary file 3 — Additional file 3: Table S3. Comparison of clinical characteristics between reduced EC and non-reduced EC groups. [file 12872_2020_1484_MOESM3_ESM.docx]

Supplemental Table 3. Comparison of clinical characteristics between reduced EC and non-reduced EC groups

|  | reduced EC (n = 93) | non-reduced EC (n = 226) | P value |
| --- | --- | --- | --- |
| Age | 68.8 ± 11.3 | 62.3 ± 11.4 | < 0.01 |
| Male (%) | 68 (73.1) | 188 (83.2) | 0.04 |
| BMI | 24.2 (3.4) | 23.2 (3.3) | 0.03 |
| Diabetes mellitus (%) | 46 (49.5) | 61 (27.0) | < 0.01 |
| Hypertension (%) | 63 (67.7) | 146 (64.6) | 0.59 |
| Dyslipidemia (%) | 56 (60.2) | 116 (51.3) | 0.14 |
| Chronic kidney disease (%) | 34 (36.6) | 40 (17.8) | < 0.01 |
| Current smoking (%) | 10 (10.9) | 34 (15.0) | 0.32 |
| COPD (%) | 8 (8.6) | 11 (4.9) | 0.20 |
| Cancer (%) | 0 (0) | 3 (1.3) | 0.43 |
| History of CVD | | | |
| MI (%) | 9 (9.7) | 26 (11.5) | 0.63 |
| PCI (%) | 27 (29.0) | 29 (12.8) | < 0.01 |
| CABG (%) | 6 (6.5) | 9 (4.0) | 0.34 |
| Valvular surgery (%) | 6 (6.5) | 10 (4.4) | 0.45 |
| CHF (%) | 24 (25.8) | 35 (15.5) | 0.03 |
| CVD at the beginning of CR | | | |
| Acute myocardial infarction (%) | 7 (7.5) | 32 (14.2) | 0.10 |
| Effort angina pectoris (%) | 8 (8.6) | 40 (17.7) | 0.04 |
| PCI (%) | 10 (10.8) | 43 (19.0) | 0.07 |
| CABG (%) | 20 (21.5) | 49 (21.7) | 0.97 |
| Valvular disease (%) | 43 (46.2) | 74 (32.7) | 0.02 |
| Valvular surgery (%) | 28 (30.1) | 69 (30.7) | 0.92 |
| Aortic disease (%) | 4 (4.3) | 19 (8.4) | 0.20 |
| Peripheral artery disease (%) | 4 (4.3) | 7 (3.1) | 0.59 |
| Atrial fibrillation (%) | 28 (30.1) | 22 (9.8) | < 0.01 |
| Anthropometric data | | | |
| Body fat percentage (%) | 26.2 ± 8.1 | 20.9 ± 7.7 | < 0.01 |
| Lean body weight (kg) | 47.9 ± 9.0 | 50.0 ± 8.4 | 0.06 |
| Trunk muscle mass (kg) | 24.2 ± 4.2 | 25.6 ± 4.0 | < 0.01 |
| Upper limb muscle mass (kg) | 4.6 ± 1.0 | 4.8 ± 1.0 | 0.14 |
| Lower limb muscle mass (kg) | 16.5 ± 3.9 | 17.0 ± 3.7 | 0.27 |
| Grip strength (kg) | 27.5 ± 8.0 | 32.7 ± 8.0 | < 0.01 |
| Echocardiography | | | |
| LVEF (%) | 56 ± 16 | 57 ± 14 | 0.40 |
| E/A | 1.4 ± 1.0 | 1.3 ± 0.8 | 0.40 |
| E/e' | 16.0 ± 8.1 | 12.5 ± 7.3 | < 0.01 |
| Laboratory data | | | |
| Hemoglobin (g/dL) | 13.0 ± 1.8 | 13.8 ± 1.7 | < 0.01 |
| Albumin (g/dL) | 3.8 ± 0.5 | 4.0 ± 0.4 | < 0.01 |
| Creatinine (mg/dL) | 1.25 ± 1.63 | 0.85 ± 0.52 | < 0.01 |
| eGFR (mL/min/1.73 m^2^) | 64.3 ± 23.9 | 77.5 ± 21.5 | < 0.01 |
| TG (mg/dL) | 128 ± 91 | 120 ± 69 | 0.40 |
| HDL cholesterol (mg/dL) | 46 ± 14 | 50 ± 16 | 0.02 |
| LDL cholesterol (mg/dL) | 99 ± 28 | 101 ± 29 | 0.63 |
| HbA1c (%) | 6.2 ± 0.7 | 5.9 ± 0.7 | < 0.01 |
| BNP (pg/nL) | 290.3 ± 674.3 | 134.2 ± 223.9 | < 0.01 |
| Skin autofluorescence (a.u) | 3.1 ± 0.7 | 2.8 ± 0.5 | < 0.01 |
| Medication | | | |
| Aspirin (%) | 66 (71.0) | 189 (84.0) | < 0.01 |
| ACE-I/ARB (%) | 45 (48.4) | 83 (36.9) | 0.06 |
| Statin (%) | 56 (60.2) | 137 (60.9) | 0.91 |
| β blocker (%) | 63 (67.7) | 169 (75.1) | 0.18 |
| Ca antagonist (%) | 20 (21.5) | 30 (13.3) | 0.07 |
| Loop diuretics (%) | 68 (73.1) | 150 (66.7) | 0.26 |
| Oral hypoglycemic agent (%) | 23 (24.7) | 25 (11.1) | < 0.01 |
| Insulin (%) | 6 (6.5) | 8 (3.6) | 0.25 |
| Anaerobic threshold (AT) | | | |
| Workload (W) | 37 ± 11 | 49 ± 15 | < 0.01 |
| AT (mL/kg/min) | 9.2 ± 1.2 | 12.0 ± 2.3 | < 0.01 |
| Peak exercise | | | |
| HR (/min) | 102 ± 21 | 117 ± 18 | < 0.01 |
| SBP (mmHg) | 164 ± 29 | 181 ± 29 | < 0.01 |
| DBP (mmHg) | 81 ± 16 | 89 ± 16 | < 0.01 |
| RER | 1.10 ± 0.12 | 1.12 ± 0.10 | 0.11 |
| Workload (W) | 64 ± 13 | 88 ± 20 | < 0.01 |
| Peak VO_2_ (mL/kg/min) | 12.3 ± 1.3 | 18.1 ± 3.0 | < 0.01 |
| VE/VCO_2_ | 33.9 ± 8.9 | 29.8 ± 5.9 | < 0.01 |

Data are presented as the mean value ± SD. BMI, body mass index; COPD, chronic obstructive pulmonary disease; CVD, cardiovascular disease; MI, myocardial infarction; PCI, percutaneous coronary intervention; CABG, coronary artery bypass graft; CHF, congestive heart failure; CR, cardiac rehabilitation; LV, left ventricular; EF, ejection fraction; E, early diastolic filling velocity; A, late diastolic filling velocity; e’, early diastolic tissue velocity; eGFR, estimate glomerular filtration rate; TG, triglyceride; HDL, high-density lipoprotein cholesterol; LDL, low-density lipoprotein cholesterol; HbA1c, hemoglobin A1c; BNP, B-type natriuretic peptide; HR, heart rate; SBP, systolic blood pressure; DBP, diastolic blood pressure; RER, respiratory exchange ratio; peak VO_2_, peak oxygen uptake.
